# Clinicopathological associations and prognostic values of IDH1 gene mutation, MGMT gene promoter methylation, and PD-L1 expressions in high-grade glioma treated with standard treatment

**DOI:** 10.11604/pamj.2020.36.309.24831

**Published:** 2020-08-20

**Authors:** Julius July, Diana Patricia, Pricilla Yani Gunawan, Handrianto Setiajaya, Teridah Ernala Ginting, Teguh Pribadi Putra, Zerlina Wuisan, Dini Budhiarko, Najmiatul Masykura, Gintang Prayogi, Ahmad Rusdan Utomo, Steven Tandean, Michael Lumintang Loe

**Affiliations:** 1 Department of Neurosurgery, Medical Faculty of Universitas Pelita Harapan, Neuroscience Centre of Siloam Hospital Lippo Village, Tangerang, Indonesia,; 2 Mochtar Riady Institute for Nanotechnology and Medical Science group, Universitas Pelita Harapan, Lippo Village, Tangerang, Indonesia,; 3 Department of Pathology, Medical Faculty of Universitas Pelita Harapan, Neuroscience Centre of Siloam Hospital Lippo Village, Tangerang, Indonesia,; 4 Department of Neurology, Medical Faculty of Universitas Pelita Harapan, Neuroscience Centre of Siloam Hospital Lippo Village, Tangerang, Indonesia,; 5 Division of Neurosurgery, Department of Surgery, Rumah Sakit Pusat Angkatan Darat Gatot Subroto, Jakarta, Indonesia,; 6 Stemcell and Cancer Institute, PT Kalbe Farma Tbk, Jakarta, Indonesia,; 7 Department of Neurosurgery, Faculty of Medicine, Universitas Sumatera Utara, Haji Adam Malik General Hospital, Medan 20155, Medan, Indonesia

**Keywords:** IDH1 mutation, MGMT methylation, PD-L1, anaplastic astrocytoma, glioblastoma multiforme

## Abstract

**Introduction:**

the objective was to evaluate the impact of IDH1 R132H mutation, MGMT methylation and PD-L1 expression in high grade glioma that received standard therapy (surgery, radiation and chemotherapy) to overall survival (OS).

**Methods:**

this is a retrospective study of 35 high grade glioma cases. Genotyping of IDH1 gene alteration on the mutation hotspot R132 (Sanger sequencing method with Applied Biosystems 3500 Genetic Analyzer), EZ DNA Methylation-Gold kit (Zymo Research) is used to study the methylation, Cell line BT549 (ATCC HTB-122) and HCT-116 (ATCC CCL-247) were used as unmethylated control and partially methylated control respectively. Anti-human PD-L1 antibody clone E1L3N^®^from Cell Signalling Technology (USA) and Rabbit XP^®^were used to see PDL-1 expression.

**Results:**

anaplastic astrocytoma cases had more MGMT promoter methylation (50%) than glioblastoma multiforme (GBM) (20%), more IDH1 R132H mutation (42%) than GBM (4.3%). Immunohistochemistry tumor proportion score method (TPS) identified 17% and 8.7% were PD-L1 positive in AA and GBM groups, respectively. Cases with IDH1 R132H mutation and MGMT methylation still showed better OS although with high PD-L1 expression.

**Conclusion:**

IDH1 R132H mutation and MGMT methylation were good prognostic markers. High expression of PD-L1 apparently might not indicate poor overall survival in the presence of IDH1 R132 mutation and MGMT methylation.

## Introduction

Gliomas are the most common central nervous system (CNS) tumors, which are classified into groups including astrocytic, oligodendroglial, oligoastrocytic and other tumors [1, 2]. Prior to 2016, WHO defined grades of malignancy based on tumors´ histology. Grade I tumors are usually well-circumscribed with a relatively good prognosis and prolonged survival [3]. Diffuse infiltrative astrocytoma is defined as grade 2, and those which show anaplasia and mitotic activity is defined as grade III (anaplastic astrocytoma). In addition, tumors that show microvascular proliferation and/or necrosis such as glioblastoma multiforme (GBM) are classified as grade IV. Since 2016, WHO has included both histological and molecular criteria for CNS tumors in order to achieve accurate diagnoses [1, 4, 5]. Glioblastoma multiforme (GBM) constitutes a lethal diagnosis, with a median survival time of 14-16 months, while 36 months is considered as long-term survival. A 26%-33% cases with two-year survival rate was achieved under optimized standard therapy for GBM includes surgical resection, followed by radiation and co-administration of temozolomide (TMZ), an oral alkylating agent [6, 7]. Surgical resection is the most effective way to increase survival of GBM patients. However, it is difficult to do it because these tumors are frequently invasive to highly specialized brain areas, such as those involved in the control of speech, motor function and senses [8, 9]. Beside WHO grading system [4], clinical finding, neurologic performance, tumour location [10] and molecular alterations [11], are used to predict the prognosis of patients.

Malignant tumor progression is accompanied by an altered molecular phenotype driven by gene mutation, DNA promoter methylation and chromosome codeletion. Novel biomarkers such as isocitrate dehydrogenase 1 gene (IDH1) and O6-methylguanine-DNA-methyltransferase (MGMT) gene methylation status have been proposed as useful tool to predict prognosis and survival of glioma patients [11]. IDH1 gene is an enzyme regulating cellular metabolism, epigenetic regulation, redox states, and DNA repair. Somatic mutation at codon 132 of IDH1 gene, is prevalent in WHO grade II or III gliomas and in the secondary glioblastomas, and it is associated with better outcome than those patients with IDH1 wild-type [12]. Soon after the discovery, the IDH1/2 mutations are associated with a relatively prolonged patient survival for glioma and glioblastoma in clinics, and has been a rationale for drug target. In addition, primary and secondary glioblastomas have been classified based on these IDH mutation entities [2, 4]. MGMT is a DNA repair protein, found in human and many prokaryotic organisms. The epigenetic silencing of the MGMT gene by promoter hypermethylation leads to loss of MGMT protein expression thus limiting the activity of glioma repair function. Active MGMT protein removes the alkyl adducts, preventing the crosslink formation, thereby causing the resistance to alkylating drugs [13, 14]. The methylation status of MGMT, rather than the protein expression itself, has been one predictor of alkylating drugs susceptibility and long-term survival in glioma patients [15-17].

One of the mechanisms for immune system against tumor is the generation of cytotoxic T lymphocytes (CTL) population that can infiltrate, bind and kill tumor cells. In normal condition, there is an immune checkpoint regulation to balance immune response to minimize damage or autoimmune reaction. One of the most studied immune checkpoint molecules is programmed death-1 ligand (PD-L1). The interaction of PD-L1 molecule with its receptor, programmed death-1 receptor (PD-1) molecule, on CTL will inactivate CTL functions [18, 19]. High expression of PD-L1 has been observed in various types of cancers as well as in glioblastoma, suggesting potential clinical intervention using immune checkpoint inhibition [6, 20, 21]. In this paper we reported the analysis of patient overall survival (OS) in associations with PD-L1, IDH1 and MGMT status in anaplastic astrocytoma and glioblastoma patient receiving standard therapy. We also sought to understand better prognosis and to assess the efficacy of standard therapy.

## Methods

The study was conducted in accordance with ethical approval from the Ethics Committee, Faculty of Medicine, Universitas Pelita Harapan/RS Siloam Lippo Village, Indonesia (No: FKUPH15022018).

**Patient population:** this retrospective study included 35 high grade glioma patients who underwent surgery at Siloam Karawaci Hospital, Jakarta from 2009 to 2015. The inclusion criteria for study were as follows: (i) Histologically diagnosed as high grade glioma (Anaplastic astrocytoma (AA) or Glioblastoma Multiforme (GBM)) according to WHO Classification; (ii) Patients were treated with Radiotherapy (RT) + Temozolomide (TMZ); (iii) Detailed clinical information at diagnosis and during follow up; (iv) Availability of tumor sample for molecular and immunohistochemistry (IHC) analysis. Any suspicious diagnosis for anaplastic oligodendroglioma was excluded from this population.

**Sample preparation:** formalin-fixed and parrafin-embbeded tissue blocks were processed for molecular and IHC analysis. Hematoxylin eosin (HE) staining were performed to examine the tumor area as well as to confirm the histological feature of sample by two experienced pathologists. For DNA extraction, the tumor area from 3 slides (of 4 μm thickness each) were carefully microdissected. The genomic DNA then extracted using QIAmp DNA Micro kit (Qiagen) according to the manufacturing protocol.

**IDH1 mutation analysis:** genotyping of IDH1 gene alteration on the mutation hotspot R132, was assesed using sanger sequencing method (Applied Biosystems 3500 Genetic Analyzer). Synthetic DNA was used as both mutant and wildtype controls (gBlocks Gene Fragment, Integrated DNA Technologies). The detailed methods and primers used for sequencing were referred to previous publications [22].

**MGMT promotor methylation analysis:** 20μL of samples genomic DNA (250 ng) of DNA was used for bisulphite modification process, using EZ DNA Methylation-Gold kit (Zymo Research). MYOD1 gene was used as positive control amplicon for bisulphite modification. The Methylation sensitive high resolution PCR approach was used to analyze the methylation status of MGMT promoter region, according to previous publication [23]. Cell line BT549 (ATCC HTB-122) and HCT-116 (ATCC CCL-247) were used as unmethylated control and partially methylated control respectively.

**PD-L1 immunohistochemistry:** we used anti-human PD-L1 antibody clone E1L3N^®^; from Cell Signalling Technology (USA), in ratio of 1: 300 with overnight incubation time. Rabbit XP^®^was then used as secondary antibody followed by counterstaining with haematoxylin and slides dehydration. Expression of PD-L1 by cells was identified based on stained cell within the cell membrane and/or on the cytoplasm.

**Statistical analysis:** Kaplan-Meier method was used to draw the survival curves. Comparison of overall survival of cases with IDH1 wild type and R132H mutation; methylated and unmethylated MGMT promoter; and PD-L1 positive and negative was compared using the log-rank test. The statistical analyses were done using SPSS 16.0 (IBM Corporation, Armonk, NY, USA). The two-sided significance level was set at P<0.05.

## Results

**Figure 1:**
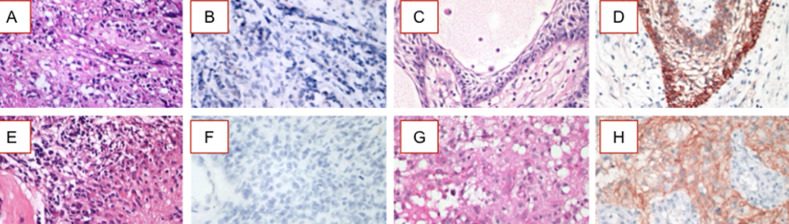
characterization of PD-L1 expressions in gliomas. Gliomas grade III (Anaplastic Astrocytoma/AA panels A to D) and grade IV (Glioblastoma Multiforme/GBM panels E to H); hematoxylin eosin images of AA (panels A and C) and GBM (panels E and G); representatives immunohistochemistry of PD-L1 negative AA (panel B) and GBM (panel F) and PD-L1 positive AA (panel D) and GBM (panel H)

**Patients characteristics:** Table 1, summarized the characteristics of the 35 patients in the study (66% males, 34% females); the median (± standard deviation) age was 51 ± 14.9 years old (range, 14 to 76 years old), under 50 years old was 17 (49%) while over 50 years old was 18 (51%). The high grade glioma in this study consists of anaplastic astrocytoma (AA) or grade III and glioblastoma (GBM) or grade IV. The AA group was 34% (12/35) and the GBM group was 66% (23/35) of patients. The molecular scoring including IDH1and MGMT showed that IDH1 wild type was 83% (29/35), IDH1 R132H mutation was 17% (6/35), MGMT methylation was 30% (7/23) and MGMT unmethylated was 70% (16/23). Immunohistochemistry scoring of PD-L1 negative expression was 88.6% (31/35) and PD-L1 positive was 11.4% (4/35) (Figure 1). The mean overall survival (OS) of all patients was 18.07±11.6 months. Sample size for all characteristics is n = 35. Sample size for MGMT methylation status was n = 23, where only patients with available MGMT data was included. *MGMT methylated patient numbers consisted of four patients with complete methylation and three with partial methylation.

**Table 1:** characteristics of glioma patients

Characteristics	Value
**Gender n (%)**	
Male	23 (66)
Female	12 (34)
**Age (year)**	
Median	51-14.9
Range	14 - 76
Age < 50	17 (49)
Age ≥ 50	18 (51)
**WHO grading system n (%)**	
AA	12 (34)
GBM	23 (66)
**Molecular alterations n (%)**	
IDH1 WT	29 (83)
IDH1 R132H	6 (17)
MGMT met	7 (30) *
MGMT unmet	16 (70)
MGMT unavailable	12
PD-L1 negative	31 (88.6)
PD-L1 positive	4 (11.4)
Overall survival (months)	18.07-11.6

* MGMT methylated patient numbers consisted of 4 patients with complete methylation and 3 with partial methylation

**Figure 2:**
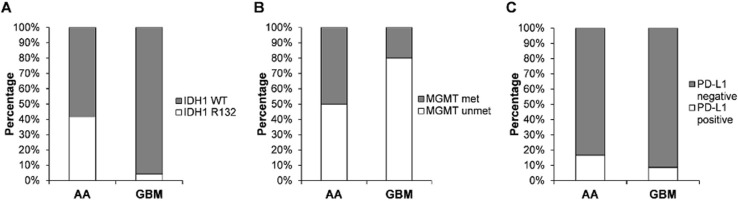
proportion of IDH1 wild type (WT) and R132H mutation (A); MGMT promotor methylation (MGMT met) and unmethylated (MGMT unmet) (B); and PD-L1 negative and PD-L1 positive (C) in anaplastic astrocytoma (AA) and glioblastoma (GBM) patients

**Frequency of IDH1 mutation, MGMT promoter methylation and PD-L1 expression status:** among a total 35 glioma cases, the frequency of IDH1, MGMT promoter methylation and PD-L1 expression status of glioma patients were represented in Figure 2. By molecular analysis, IDH1 R132H mutations were identified in five of twelve cases (42%) in the AA group, and one of twenty three cases (4.3%) in GBM group. Of a total thirty five cases, twelve cases were unable to be identified, thus only 23 cases were reported in this paper. MGMT promoter methylation was identified in four of eight cases (50%) in the AA group and three of twelve cases (20%) in the GBM. Of four MGMT methylated cases, three cases were partially methylated and were included in AA group. Tumor proportion score (TPS) value of PD-L1 expression showed two of twelve cases (17%) and two of twenty three cases (8.7%) were PD-L1 positive in AA and GBM groups, respectively. Of all cases, two cases were identified with both IDH1 R132H mutation and MGMT promotor methylation, and one of them was PD-L1 positive (< 10% TPS) (Table 2). Age of cases with both IDH1 R132H mutation and MGMT promotor methylation were over 50 years old and among AA group. Survival time for both cases were 42 and 32 months.

**Table 2:** PD-L1 status of cases with the presence of IDH1 R132H mutation and MGMT promoter methylation

No.	Age	WHO grades	Survival (month)	PD-L1 TPS method
1	≥ 50 years	AA	42	Negative
2	≥ 50 years	AA	32	<10%

**Figure 3:**
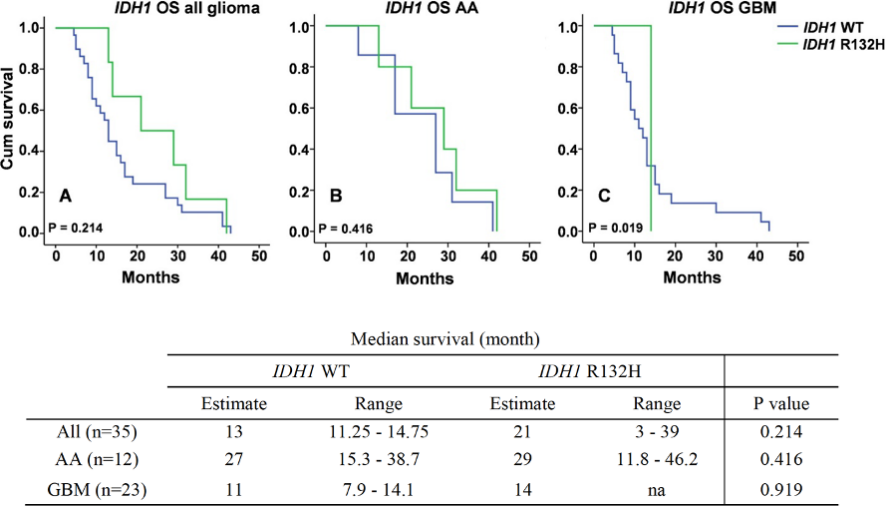
survival of glioma patients based on IDH1 mutation status in all cases (A), anaplastic astrocytoma (AA) (B) and glioblastoma (GBM) (C) cases. IDH1 WT = IDH1 wild type, IDH1 R132H = IDH1 mutation arginine to histidine at amino acid 132

**Overall survival of all glioma, AA and GBM cases based on IDH1 gene mutation:** the overall survival based on IDH1 mutation status is summarized in Figure 3. Of a total 35 glioma patients, the estimated Kaplan-Meier survival with IDH1 wild type (IDH1 WT) was 13 months (range 11.25 to 14.75 months), while patients with IDH1 R132H mutation (IDH1 R132H) was 29 months (range 11.8 to 46.2 months). Survival in AA group with IDH1WT and IDH1 R132H were 27 months (range 15.3-38.7) and 29 months (range 11.8-46.2), respectively. For GBM group, the survival of patients with IDH1 WT was 11 months (range 7.9-14.1). The IDH1 R132H mutation in GBM group was found in only one patient with 14 months survival. In general, overall survival was better in cases with IDH1 R132H mutation in all glioma cases and both AA and GBM although the P values were not statistically significant (P > 0.05).

**Figure 4:**
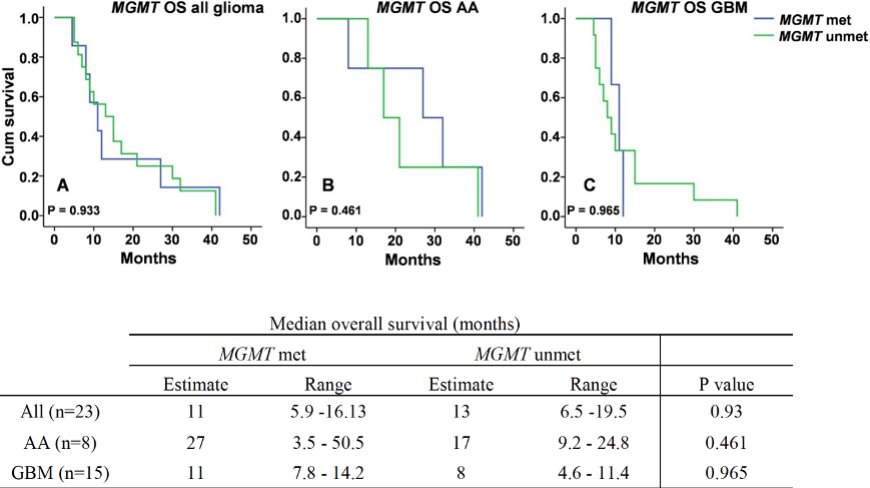
survival of glioma patients based on MGMT methylation status in all cases (A), anaplastic astrocytoma (AA) (B) and glioblastoma (GBM) (C). MGMT met = MGMT methylation, MGMT unmet = MGMT unmethylated

**Overall survival of all glioma, AA and GBM cases based on MGMT methylation:** median overall survival based on MGMT methylation was derived from cases with available MGMT methylation status data, which was 23 cases. Kaplan-Meier survival of MGMT met group in all glioma cases was 11 months (range 5.9 to 16.13 months) while for MGMT unmet group was 13 months (range 6.5 to 19.5 months) with no significant difference between groups (P > 0.05). Among the AA group, survival of cases with MGMT met was 27 months (range 3.5 to 50.5) while cases with MGMT unmet was 17 months (range 9.2 to 24.8). For GBM group, cases with MGMT met showed median survival at 11 months (7.8 to 14.2), while cases with MGMT unmet showed median survival at 8 months (range 4.6-11.4). Although MGMT methylation indicated a better survival in both AA and GBM cases when compared to the unmethylated groups, it was not statistically significant (Figure 4).

**Figure 5:**
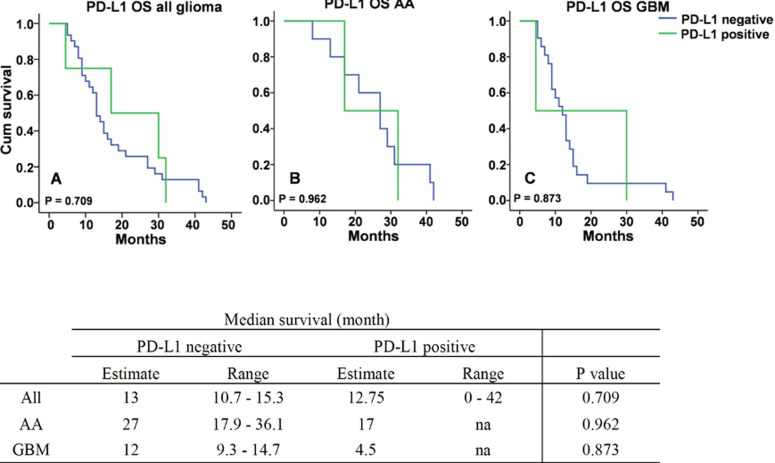
survival of glioma patients based on PD-L1 expression in all cases (A), anaplastic astrocytoma (AA) (B) and glioblastoma (GBM) (C)

**Overall survival of all glioma, AA and GBM cases based on PD-L1 expression:** the expression of PD-L1 was measured by tumor proportion scoring or TPS. Overall survival of cases with PD-L1 negative was 13 months (range 10.7-15.3), while cases with PD-L1 positive was 12.75 months (range 0 to 42). Both AA and GBM groups showed better survival when PD-L1 expression was negative (estimate survival was 27 months within the range 17.9-36.1 months and 12 months within the range 9.3-14.7, respectively) when compared to PD-L1 positive group (estimate survival was 17 and 4.5 months, respectively), although the difference was not significant. Number of cases with PD-L1 positive in both AA and GBM groups (only 2 patients for each group) were relatively low in this study thus range of survival was not applicable (Figure 5).

## Discussion

Glioma is a malignant tumor with extremely poor prognosis. Currently, the main therapy for glioma is a comprehensive treatment including surgical resection, radiotherapy and chemotherapy [9, 23-26]. Although low grade glioma patients have better survival than patients with high grade (WHO grade III/IV) glioma, all low grade gliomas eventually progress to high grade glioma and death. Molecular markers such as IDH1 mutation, MGMT methylation and PD-L1 are among the most recent and widely studied tools for diagnosis and treatment guidance [22, 27]. In this study we performed analysis of overall survival for high grade glioma (anaplastic astrocytoma/grade III and glioblastoma / grade IV) that received same standard treatment (surgical resection, radiation and temozolomide) based on its prognostic marker; IDH1 mutation, MGMT methylation and PD-L1 expression.

Previous studies showed that IDH1 R132H mutation was present in 80% of WHO grade II and III gliomas but less than 5% of primary glioblastoma. Our result showed that 42% of AA group, while only 4.3% of GBM group, possessed IDH1 R132H mutation. High percentage of IDH1 mutation in AA group is consistent with the former finding that IDH gene 1 or 2 is mutated in the vast majority of WHO grade II or III gliomas [12, 28]. Therefore, a case of GBM with IDH1 mutation in our study suggest that the tumor has developed from a lower grade (secondary GBM). Both AA and GBM groups in our study with IDH1 mutation showed better survival when compared to IDH1 wild type group. This is probably because mutation in IDH1 gene may disrupt the function of IDH1 enzyme as the main catalyst for cellular metabolism, redox state and DNA repair in tumor cells, thus leading to the less progressive tumor and better survival of patients. On the other hand, as IDH 1 and 2 have roles in oncogenesis, their occurrence prompted the development of IDH1/2-mutant inhibitors such as Enasidenib [29, 30].

Methylation of MGMT promoter is a commonly observed change as molecular prognostic factor and also predicts a favourable response to alkylating agent chemotherapy [31-33]. On the basis of our data, MGMT promoter methylation is more frequent in AA cases when compard to GBM (50% and 20%, respectively). It appears that MGMT promoter methylation in both AA and GBM groups gives better survival when compared to those of without MGMT promoter methylation, points its important role as prognostic factor in glioma cases. Part of the aggressive nature of glioma is related to its ability to escape immune system surveillance by an overexpression of immune checkpoint molecules such as PD-L1. Thus, the status of PD-L1 expression is important for considering therapy combination with immune checkpoint molecule blockade drugs. The rate of PD-L1 positive cases in our study seems relatively low in both AA and GBM groups (17% and 8.7%, respectively) when compared with glioma and solid tumor types in other studies [20, 34]. In our present study, cases tested positive PD-L1 seems to have shorter survival of both AA and GBM cases. However, there are only 4 PD-L1 positive cases (2 for each groups) in this study which may not enough to represent overall glioma cases.

We also report 2 AA cases within the similar median age (both with age over 50 years), both possessed good prognostic markers ie positive IDH1 R132H mutation and MGMT promoter methylation. However, one case was with negative PD-L1 and the other was positive PD-L1. In consequence of the good prognostic markers, both cases seem to have relatively long survival rate (over two years), although case with PD-L1 positive appears to have shorter survival in comparison to that of with negative PD-L1 status (42 months and 32 months, respectively). Based on literatures, studies on the prognostic impact of tumoral PD-L1 expression have shown inconsistent results across tumor types [35-37]. In our series including a total of 35 glioma cases, here we provide evidence of high grade glioma case with positive IDH1 R132H mutation and MGMT promoter methylation that reached the long-term survival rate (over 36 month) when the PD-L1 expression status is negative, in comparison to its positive PD-L1 counterpart. In addition, the histological grade of glioma still the main indicator of patient survival, ie grade III glioma has longer survival than grade IV glioma, regardless to their molecular prognostic marker and PD-L1 status. Our study is limited by the sample size and our results surely need confirmation in larger cohort.

## Conclusion

This study shows that PD-L1 is detectable in minority of glioma samples, suggesting that the immune checkpoint blockade therapy would be a benefit for those patients. Molecular markers such as IDH1 R132H mutation and MGMT methylation are relevant for good prognostic marker in our data. High expression of PD-L1 apparently does not indicate a worse overall survival when IDH1 R132 mutation and MGMT methylation are positive. The survival rate is likely associated with histological grade of the glioma. Taken together, standardized glioma treatment is still relevant for high grade glioma management in respect of overall survival analysis.

### What is known about this topic

- Glioblastoma multiforme (GBM) constitutes a lethal diagnosis, with a median survival time of 14-16 months, while 36 months is considered as long-term survival;
- Standard therapy for GBM includes surgical resection, followed by radiation and co-administration of temozolomide (TMZ), an oral alkylating agent;
- Beside WHO grading system, clinical finding, neurologic performance, tumour location and molecular alterations, are used to predict the prognosis of patients.

### What this study adds

- PD-L1 is detectable in minority of glioma samples, suggesting that the immune checkpoint blockade therapy would be a benefit for those patients; IDH1 R132H mutation and MGMT methylation are relevant for good prognostic marker;
- High expression of PD-L1 apparently does not indicate a worse overall survival when IDH1 R132 mutation and MGMT methylation are positive;
- Standardized glioma treatment is still relevant for high grade glioma management in respect of overall survival analysis.
